# Prevalence of diabetes among Indigenous women in Guatemala: a retrospective chart review

**DOI:** 10.1186/s13104-024-06838-0

**Published:** 2024-07-12

**Authors:** Stephen Alajajian, Jenny Bartolimin, Yolanda Juarez Martin, Caitlin Scott, Peter Rohloff, David Flood

**Affiliations:** 1 Centro de Investigación en la Salud Indígena, Wuqu’ Kawoq, 2da Avenida 3-48 Zona 3 Barrio Patacabaj, Tecpán, Chimaltenango, Guatemala; 2 Friendship Bridge, 405 Urban St. Ste. 140, Lakewood, CO USA; 3 Division of Global Health Equity, Brigham and Women’s Hospital, 75 Francis St, Boston, MA USA; 4 Department of Internal Medicine, University of Michigan, 500 S State St, Ann Arbor, MI USA

## Abstract

**Objective:**

The objective of this study is to investigate the prevalence of diabetes in a clinical population of primarily Indigenous women in Guatemala.

**Results:**

In a retrospective chart review of a clinical program serving 13,643 primarily Indigenous women in Guatemala, crude diabetes prevalence was 8.3% (95% Confidence Interval [CI]: 7.8 to 8.7) and age-adjusted diabetes prevalence was 7.9% (95% CI: 7.3 to 8.5). Among those with diabetes, 37.9% (95% CI: 35.1 to 40.8) of women were undiagnosed. Diabetes prevalence rose significantly with increasing age and was significantly higher among women with obesity (risk ratio: 1.4 [95% CI: 1.1 to 1.8]) and among women least likely to be in poverty (risk ratio: 2.0 [95% CI: 1.5 to 2.6]). Diabetes prevalence was significantly lower among Indigenous women (risk ratio: 0.7 [95% CI: 0.6 to 0.9]) and among women who spoke Mayan languages rather than Spanish (risk ratio: 0.7 [95% CI: 0.6 to 0.9]). There was no significant difference in diabetes prevalence between women who lived in rural settings and women who lived in urban settings.

## Introduction

Guatemala is a middle-income Central American nation with a total land area of 108.9 square kilometers and a majority-rural population of 18.3 million people, nearly half of whom are Indigenous [1, 2]. The population of Guatemala faces significant health and development issues which include high prevalence of poverty and malnutrition, scarce employment opportunities and limited access to healthcare and education [2]. These issues disproportionately affect the Indigenous population, the majority of whom are ethnically Maya [2]. 

Modeling studies suggest that the diabetes burden is rising rapidly in Guatemala [3, 4]. Rising diabetes prevalence in Guatemala as in other countries with developing economies may reflect changes in dietary and physical activity patterns related to urbanization, shifts in agricultural practices and increased availability of refined food [5]. Like other Latin American countries with large numbers of Indigenous people, in Guatemala there is limited data on the epidemiology of diabetes in this marginalized population. The objective of this study is to investigate the prevalence of diabetes in a clinical population of primarily Indigenous women in Guatemala.

## Methods

### Study design and program description

We conducted a retrospective chart review using data from a non-governmental, community-based primary care program serving adult women from a microfinance program in nine Central and Western departments in Guatemala. Program details previously have been described [6, 7]. Study size was limited by the number of available medical records for which glucose data was available. Upon enrollment in the program, patients were queried if they had a history of diabetes and, if not, were screened for diabetes using random or fasting glucose capillary measurements.

### Data extraction and definition of diabetes

For this analysis, we extracted electronic health record and administrative data between July 1, 2015, and December 31, 2022. We defined diabetes by self-reported prior history, current use of a glucose-lowering medication, or an elevated blood glucose (fasting ≥ 126 mg/dl or random ≥ 200 md/dl) using the last available recorded glucose value.

### Data analysis

We calculated crude and age-adjusted diabetes prevalence overall and by the following patient characteristics: age group, body mass index (BMI) category, ethnicity (Indigenous or non-Indigenous), language (Spanish or Mayan), residence (rural or urban), and economic status. We age-standardized estimates to the World Health Organization (WHO) reference population [8]. Age-adjustment was used to account for age-related bias. Economic status was assessed using the Poverty Probability Index (PPI), which uses asset ownership to predict the probability that a household was below the national poverty line [9]. PPI probabilities were grouped into quartiles. Absolute differences and relative differences (as risk ratios) in diabetes prevalence were computed within levels of each characteristic. We used multiple imputation with chained equations to impute missing data on patient characteristics (50 iterations). Results were reported with logit-transformed 95% confidence intervals [10]. We used Stata version 17.

## Results

The final analytic dataset included 13,643 patients after excluding those with no glucose data recorded (*n* = 387) and without a legitimate medical record number (*n* = 3). Among the patient observations, the last available glucose was fasting glucose for 2,172 women (16.0%) and random glucose for 11,471 women (84.1%). The median age was 40 years (interquartile range [IQR]: 31–51). The median BMI was 27.3 kg/m^2^ (IQR: 24.1–30.9); 38.7% were overweight (*n* = 5,206), and 30.2% were obese (*n* = 4,065). Patients were predominantly Indigenous (90.3%) (*n* = 12,311) and lived in rural areas (85.1%) (*n* = 6,510). Overall, 44.4% were estimated to be living in poverty based on PPI probabilities (*n* = 6,057). Missing data was high for area of residence (43.9%) (*n* = 5,995) and economic status (42.3%) (*n* = 5,772).

**Table 1:** Demographic characteristics of a cohort of 13,643 primarily Indigenous women in Guatemala

	Total (*n* = 13,643) median (inter-quartile range) *n* (%)	Diabetes (*n* = 1,126)	No diabetes (*n* = 12,517)	Missing *n* (%)
Age, median (years) (IQR)	40 (31–51)	53 (44–59)	39 (31–49)	4 (0.0)
Number of children, median (IQR)	3 (2–5)	5 (3–7)	3 (2–5)	591 (4.3)
BMI, median (kg/m^2^) (IQR)	27.3 (24.1–30.9)	28.3 (25.2–31.8)	27.2 (24.1–30.8)	202 (1.5)
BMI Class				202 (1.5)
Underweight (BMI < 18.5)	140 (1.0)	9 (1.0)	131 (1.1)	
Normal weight (BMI 18.5–24.9)	4,030 (30.0)	247 (22.4)	3,783 (30.7)	
Overweight (BMI 25-29.9)	5,206 (38.7)	447 (40.6)	4,759 (38.6)	
Obese (BMI > = 30)	4,065 (30.2)	399 (36.2)	3,666 (29.7)	
Ethnicity				2 (0.0)
Indigenous	12,311 (90.3)	978 (86.7)	11,335 (90.6)	
Not indigenous	1,330 (9.8)	150 (13.3)	1,180 (9.4)	
Language				2,765 (20.3)
Mayan language	6,425 (59.1)	503 (54.2)	5,922 (59.5)	
Spanish	4,453 (40.9)	425 (45.8)	4,028 (40.5)	
Residence				5,995 (43.9)
Rural	6,510 (85.1)	595 (84.0)	5,915 (85.2)	
Urban	1,138 (14.9)	113 (16.0)	1,025 (14.8)	
Economic Status^1^ (Quartiles)				5,772 (42.3)
1 (Most likely in poverty)	2,098 (26.7)	119 (16.8)	1,979 (27.6)	
2	1,860 (23.6)	155 (21.9)	1,705 (23.8)	
3	2,478 (31.5)	235 (33.2)	2,243 (31.3)	
4 (Least likely in poverty)	1,435 (18.2)	198 (28.0)	1,237 (17.3)	

^1^As assessed by Poverty Probability Index (PPI)

Across all patient observations, there were 1,126 women with diabetes. The crude diabetes prevalence was 8.3% (95% Confidence Interval [CI]: 7.8 to 8.7), and the age-adjusted diabetes prevalence was 7.9% (95% CI: 7.3 to 8.5). Among those with diabetes, 62.1% (95% CI: 59.2 to 64.5) of women self-reported a prior diagnosis (*n* = 699) and 37.9% (95% CI: 35.1 to 40.8) of women were newly diagnosed (*n* = 427).

By patient characteristics, diabetes prevalence rose sharply with increasing age, reaching nearly 20% in women who were 50 years of age or older. Compared to the reference of normal BMI, women who were obese had an age-standardized diabetes prevalence that was 3.2% (95% CI: 1.1 to 5.2) greater in absolute magnitude and approximately 40% greater in relative magnitude (risk ratio: 1.4 [95% CI: 1.1 to 1.8]). Age-standardized diabetes was higher among non-Indigenous versus Indigenous women and among Spanish versus Mayan language speakers. Compared to those most likely to be in poverty, women least likely to be in poverty had an age-standardized diabetes prevalence that was 6.0% (95% CI: 3.7 to 8.3) greater in absolute magnitude and 100% greater in relative magnitude (risk ratio: 2.0 [95% CI: 1.5 to 2.6).

**Fig. 1:**
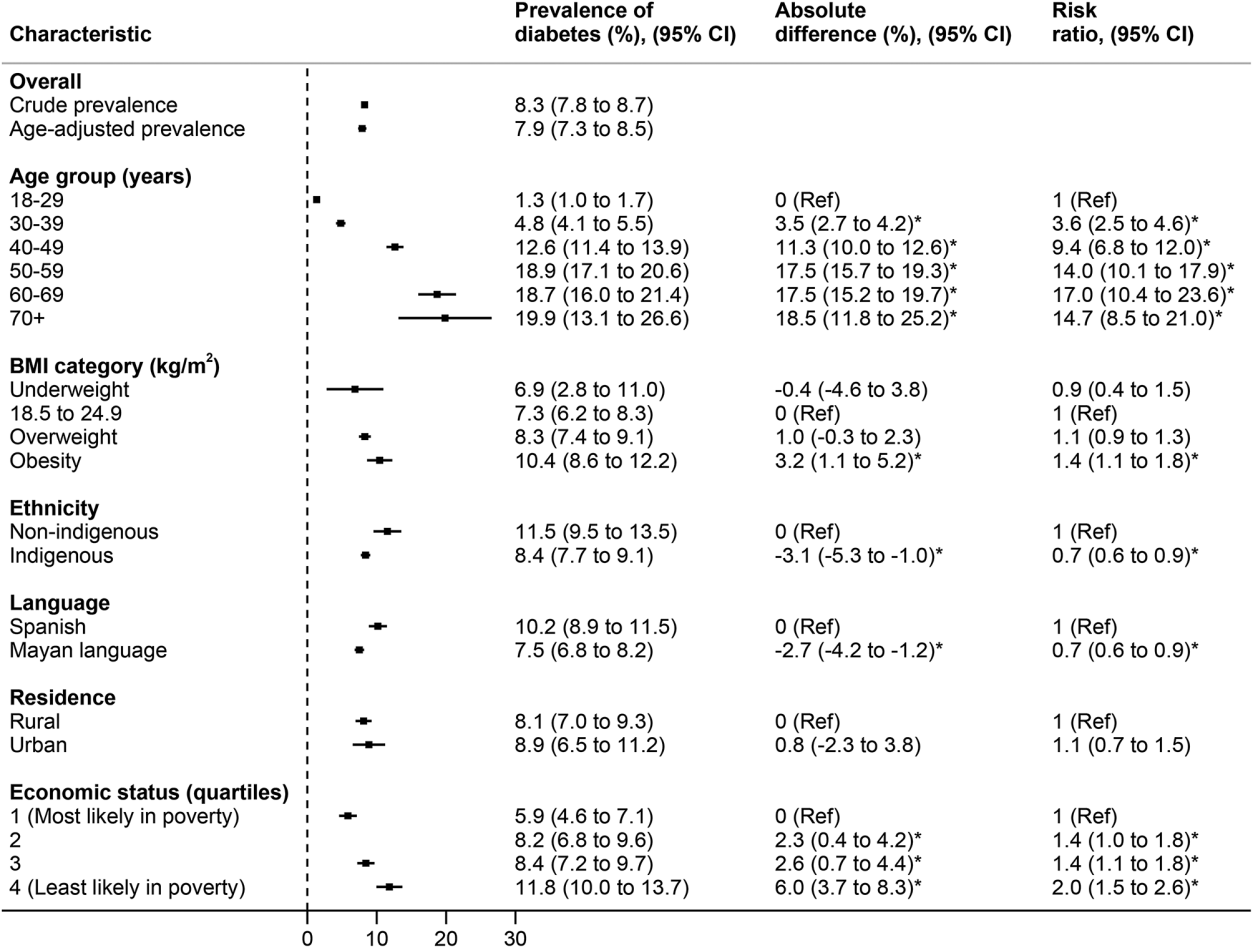
Prevalence of diabetes among a cohort of 13,643 primarily Indigenous women in Guatemala, overall and by demographic characteristic Footnote: For economic status quartiles, the probabilities of poverty were as follows: quartile 1, ≥78.2%; quartile 2, ≥46.6% to <78.1%; quartile 3, ≥15.6% to <46.5%; quartile 4, <15.6%. Statistically significant results are indicated with an asterisk (*)

## Discussion

In a retrospective chart review of a clinical program serving 13,643 primarily Indigenous women in Guatemala, we found that the age-adjusted diabetes prevalence was 7.9% (*n* = 1,126) and that approximately 37.9% of people with diabetes were undiagnosed (*n* = 427). Diabetes prevalence was significantly higher in older women, women with obesity, non-Indigenous women, speakers of Spanish rather than Mayan languages and those least likely to be in poverty.

To our knowledge, this study leverages the largest programmatic or survey dataset of primarily Indigenous people with diabetes in Guatemala. A few prior household surveys conducted in select predominantly Indigenous areas have reported diabetes prevalence ranging from 3.0 to 13.5% [11–13]. Differences in these survey estimates may be explained by variations in diabetes definition, time period of assessment, or differences in the underlying population. A national survey conducted from 2018 to 2019 among approximately 2,500 reproductive aged women aged 15–49 years reported diabetes prevalence of approximately 6% [14]. Unfortunately, to date, there is no other Guatemalan national health survey—such as a WHO STEPS survey—that includes diabetes assessments.

Diabetes prevalence in this clinical population differed significantly across patient characteristics. Increased prevalence in those least likely to be in poverty may reflect shifts in dietary and physical activity patterns associated with greater affluence. For Mayan language speakers and Indigenous women, a strong sense of cultural identity could be protective against diabetes [15]. Lower diabetes prevalence in these groups might also reflect cultural differences in dietary and lifestyle patterns, or differences in income levels. We found obesity to be associated with a 40% increase in diabetes prevalence in this primarily rural and Indigenous Guatemalan population, contrary to Bream et al. who found no association between BMI and diabetes prevalence [12].

## Limitations

The main limitation of the study is that our patient population is restricted to women and was not drawn from a representative sampling frame, limiting generalizability. At the same time, this clinical population is quite similar in age structure, language preference, and economic status to the overall Indigenous population of women in these geographic areas of Guatemala [16]. Another limitation is the absence of hemoglobin A1c data in this resource limited context, which make comparisons of diabetes prevalence with studies that use hemoglobin A1c data more difficult. The strength of the current study is our large number of observations and use of repeat testing to confirm diabetes among newly diagnosed women with elevated random glucose. The large n gives us added power to investigate differences in diabetes prevalence across meaningful subgroups of the population.

## Conclusion

In conclusion, we report age-adjusted diabetes prevalence of 7.9% in a large patient population of Indigenous women living in Guatemala. Our data reinforce the need to strengthen the rural health system in Guatemala to deliver high-quality care not only for maternal and child health conditions but also non-communicable diseases such as diabetes.

## Data Availability

The datasets generated during and/or analyzed during the current study are not publicly available as they are proprietary data of the collaborating microfinance institution but may be made available from the corresponding author with permission from the institution upon reasonable request. Statistical code is available at the Harvard Dataverse (10.7910/DVN/GA8JEA).
